# Supplementary material for: Development and Validation of a New Measure of Work Annoyance Using a Psychometric Network Approach
Source: Int J Environ Res Public Health. 2022 Jul 30;19(15):9376. doi: 10.3390/ijerph19159376 (PMC9368152; doi:10.3390/ijerph19159376)
Supplement: Supplementary file 1 [file ijerph-19-09376-s001.zip › ijerph-1805983-supplementary.pdf]

International Journal of
Environmental Research and Public Health

MDPI

Supplementary Materials for the article:
Development and validation of a new measure of work annoyance using a psychometric network approach

Nicola Magnavita [1] and Carlo Chiorri [2]*

1. Correlation matrices of Work Annoyance Scale items

As explained in Section 2.4 of the manuscript, we computed the correlation matrix of the WAS items in both cohorts with and without taking into account the nesting of observations into organizations. We report in tables S1 and S2 these matrices. The Steiger's [1] test for difference of the correlation matrices, performed using the *cortest.normal* function in the R package *psych* [2], was not significant in either case (Cohort 1: $\chi^2(36) = 16.38$, $p = .998$; Cohort 2: $\chi^2(36) = 3.81$, $p > .999$).

Table S1. Coefficients of the zero-order (lower triangle) and of within correlation matrix (upper triangle), and intraclass correlation coefficients (ICC) for the Work Annoyance Scale items from data of Cohort 1 (n = 2,226).

	was01	was02	was03	was04	was05	was06	was07	was08	was09
was01	**.064**	.381	.198	.370	.187	.149	.355	.404	.317
was02	.390	**.119**	.188	.323	.189	.159	.390	.387	.224
was03	.221	.199	**.021**	.269	.729	.486	.271	.111	.495
was04	.406	.331	.287	**.106**	.267	.234	.377	.376	.294
was05	.203	.197	.732	.280	**.009**	.555	.282	.142	.460
was06	.164	.158	.488	.238	.555	**.021**	.250	.107	.342
was07	.383	.400	.288	.402	.293	.258	**.048**	.508	.350
was08	.431	.398	.135	.397	.156	.123	.525	**.053**	.269
was09	.350	.236	.505	.319	.464	.354	.373	.303	**.045**

Note: bolded values on the diagonal are ICCs.

Table S2. Coefficients of the zero-order (lower triangle) and of within correlation matrix (upper triangle), and intraclass correlation coefficients (ICC) for the Work Annoyance Scale items from data of Cohort 2 (n = 665).

	was01	was02	was03	was04	was05	was06	was07	was08	was09
was01	**.038**	.424	.311	.379	.259	.247	.477	.450	.385
was02	.377	**.091**	.303	.385	.215	.214	.493	.437	.274
was03	.300	.320	**.020**	.327	.730	.494	.337	.246	.512
was04	.401	.367	.327	**.058**	.271	.255	.483	.472	.293
was05	.259	.240	.734	.285	**.028**	.552	.282	.198	.481
was06	.235	.214	.501	.247	.552	**.018**	.348	.242	.392
was07	.478	.484	.344	.499	.302	.349	**.036**	.583	.440
was08	.437	.462	.263	.471	.224	.240	.583	**.060**	.350
was09	.396	.252	.511	.312	.481	.395	.446	.341	**.022**

Note: bolded values on the diagonal are ICCs.

References

87. Revelle, W. Psych: Procedures for Psychological, Psychometric, and Personality Research Available online: http://cran.r-project.org/package=psych. (accessed on 15 April 2022).

111. Steiger, J.H. Testing Pattern Hypotheses on Correlation Matrices: Alternative Statistics and Some Empirical Results. *Multivariate Behav. Res.* **1980**, *15*, 335–352,

doi:10.1207/s15327906mbr1503_7.

Supplementary File S1 - The Work Annoyance Scale

Italian version

1. Quanto ti dà fastidio il fatto di dover lavorare oltre l'orario stabilito (o ti darebbe fastidio se dovessi farlo)?

Nessun fastidio	0	1	2	3	4	5	6	7	8	9	10	Massimo Fastidio

2. Quanto ti dà fastidio il fatto di dover lavorare di notte (o ti darebbe fastidio se dovessi farlo)?

Nessun fastidio	0	1	2	3	4	5	6	7	8	9	10	Massimo Fastidio

3. Quanto ti dà fastidio il fatto di dover apprendere nuove tecniche per fare il tuo lavoro (o ti darebbe fastidio se dovessi farlo)?

Nessun fastidio	0	1	2	3	4	5	6	7	8	9	10	Massimo Fastidio

4. Quanto ti dà fastidio il fatto di doverti spostare per lavoro (o ti darebbe fastidio se dovessi farlo)?

Nessun fastidio	0	1	2	3	4	5	6	7	8	9	10	Massimo Fastidio

5. Quanto ti dà fastidio il fatto di dover imparare l'uso di un nuovo dispositivo elettronico (o ti darebbe fastidio se dovessi farlo)?

Nessun fastidio	0	1	2	3	4	5	6	7	8	9	10	Massimo Fastidio

6. Quanto ti dà fastidio il fatto di dover imparare una lingua straniera (o ti darebbe fastidio se dovessi farlo)?

Nessun fastidio	0	1	2	3	4	5	6	7	8	9	10	Massimo Fastidio

7. Quanto ti dà fastidio il fatto di dover fare un lavoro fisicamente gravoso (o ti darebbe fastidio se dovessi farlo)?

Nessun fastidio	0	1	2	3	4	5	6	7	8	9	10	Massimo Fastidio

8. Quanto ti dà fastidio il fatto di dover lavorare in un ambiente stressante (o ti darebbe fastidio se dovessi farlo)?

Nessun fastidio	0	1	2	3	4	5	6	7	8	9	10	Massimo Fastidio

9. Quanto ti dà fastidio doversi impegnare per risolvere un problema di lavoro (o ti darebbe fastidio se dovessi farlo)?

Nessun fastidio	0	1	2	3	4	5	6	7	8	9	10	Massimo Fastidio

English version

Note: This translation that has been provided with the sole purpose of enabling readers who do not know Italian to understand the content of the items. Although the translation of the questions was carefully crafted with the help of artificial intelligence services and reviewed by the authors, before using the English version of the scale, it is necessary to verify the comprehensibility of the questions for a native English-speaking audience and test its psychometric properties.

1. How much does it annoy you that you have to work beyond the fixed hours (or would annoy you if you had to)?

No annoyance	0	1	2	3	4	5	6	7	8	9	10	Utmost annoyance

2. How much does it annoy that you have to work at night (or would dislike it if you had to)?

No annoyance	0	1	2	3	4	5	6	7	8	9	10	Utmost annoyance

3. How much does it annoy you that you have to learn new techniques to do your job (or would annoy you if you had to)?

No annoyance	0	1	2	3	4	5	6	7	8	9	10	Utmost annoyance

4. How much does it annoy you that you have to travel for work (or would annoy you if you had to)?

No annoyance	0	1	2	3	4	5	6	7	8	9	10	Utmost annoyance

5. How much does it annoy you having to learn how to use a new electronic device (or would annoy you if you had to)?

No annoyance	0	1	2	3	4	5	6	7	8	9	10	Utmost annoyance

6. How much does it annoy you having to learn a foreign language (or would annoy you if you had to)?

No annoyance	0	1	2	3	4	5	6	7	8	9	10	Utmost annoyance

7. How much does it annoy you that you have to do physically demanding work (or would annoy you if you had to do it)?

No annoyance	0	1	2	3	4	5	6	7	8	9	10	Utmost annoyance

8. How much does it annoy you that you have to work in a stressful environment (or would annoy you if you had to)?

No annoyance	0	1	2	3	4	5	6	7	8	9	10	Utmost annoyance

9. How much does it annoy you to have to work hard to solve a work problem (or would it annoy you if you had to do it)?

No annoyance	0	1	2	3	4	5	6	7	8	9	10	Utmost annoyance

Supplementary File S2 - Relevant *R* code used for the analyses

```
################################################################
##Comparison of multilevel and zero-order correlation matrices#
################################################################

#multilevel correlation matrix
mlvl <- correlation::correlation(data, multilevel = TRUE)$r

#zero-order correlation matrix
cor <- psych::cor(data)

psych::cortest.normal(mlvl,cor,n1=2226,n2=2226)

#compute ICCs
misty::multilevel.icc(data[, c(1:9)],
                         type = 1,
                         cluster = data$Cluster,
                         method = "lme4",
                         REML = FALSE)

################################################################
##Dimensionality analyses#######################################
################################################################
psych::fa.parallel(data, fa="pc", main="") #parallel analysis
psych::vss(data)$map #MAP


################################################################
##Exploratory Structural Equation Modeling#####################
################################################################
library(lavaan)
esem_model <- "
  efa('block1')*F1 =~ T1 + T2 + T3 + T4 + T5 + T6 + T7 + T8 + T9
  efa('block1')*F2 =~ T1 + T2 + T3 + T4 + T5 + T6 + T7 + T8 + T9
  T1 ~ 1
  T2 ~ 1
  T3 ~ 1
  T4 ~ 1
  T5 ~ 1
  T6 ~ 1
  T7 ~ 1
  T8 ~ 1
  T9 ~ 1
"

esem <- sem(model = esem_model, data = data, rotation = "geomin",
 estimator = "MLR", information = "observed",
      rotation.args = list(rstarts = 30, row.weights = "none",
                           algorithm = "gpa", orthogonal = FALSE,
                           std.ov = TRUE,
                           geomin.epsilon = 0.0001))



################################################################
##Exploratory Graph Analysis (EGA)#############################
################################################################

#redundancy
uva.results <- UVA(data = data, method = "wTO", type = "adapt")
```

```
uva.results$redundancy$redundant

#EGA
ega <- EGA(data, model = "glasso", algorithm = "louvain")

#Bootstrapped EGA
boot <- bootEGA(data,
                iter = 10000,
                algorithm = "louvain",
                model = "glasso",
                type = "parametric",
                plot.typicalStructure = FALSE)

sc <- dimensionStability(boot)

# Print structural consistency
sc$dimension.stability
sc$dimension.stability$structural.consistency
sc$dimension.stability$average.item.stability

# Item stability statistics plot
sc$item.stability$mean.loadings
sc$item.stability$item.stability

#Weighted composite scores from EGA

ega_weights <- as.matrix(net.loads(ega)) #gets the weights

ega_weights <- ega_weights[ order(row.names(ega_weights)), ] #or-
ders the weight matrix like the items

weighted_Scores <- data %*% ega_weights

################################################################
##Functions for standardizing variables########################
################################################################

metric <- function(variable){
  variable2 <- (variable - mean(variable, na.rm=T))/(2*sd(variable,
na.rm=T))
  return(variable2)
}

ordinal <- function(variable){
  variable2 <- qnorm((rank(variable, ties.method= "average",
na.last="keep")*2-1)/(2*length(variable)))/2
  return(variable2)
}

dichotomous <- function(variable){
  variable2 <- variable-mean(variable, na.rm=T)
  return(variable2)
}

################################################################
##Network analysis for external validity#######################
################################################################

library(GGMnonreg)
#performs the analysis with 10000 boostrap replications
netw <- ggm_inference(data, boot = TRUE,
```

```
                    B = 10000, progress = FALSE)

#gets the predictability (R-squares) for each variable
preddf <- data.frame(unclass(predictability(netw)))

#gets the residual correlation matrix
netw_mat <- netw$wadj

#assigns variable names to columns and rows of the residual
#correlations
colnames(netw_mat) <- colnames(data)
rownames(netw_mat) <- colnames(netw_mat)

#flattens the residual correlation matrix
vector <-
data.frame(row=rownames(netw_mat)[row(netw_mat)[up-
 per.tri(netw_mat)]],
col=colnames(netw_mat)[col(netw_mat)[upper.tri(netw_mat)]],
corr=netw_mat[upper.tri(netw_mat)])

#adds the confidence intervals for the residual correlations
n_res <- data.frame(cbind(vector, confint(netw)))

#creates a new variable in which residual correlations whose
#95% confidence interval does not contain zero are considered,
#while being coded as zero otherwise, in order to prepare the data
#for plotting Figures 5 and 6 in the manuscript
n_res$r <- ifelse(n2res$X2.5.*n2res$X97.5.>0, n_res$corr, 0)
```
